# Evaluating the Usability of a Remote Ischemic Conditioning Device for Pre-Hospital Stroke Management: Insights from Paramedic Simulations

**DOI:** 10.3390/neurolint16060105

**Published:** 2024-11-09

**Authors:** Bogna Anna Drozdowska, Kaden Lam, Cody Doolan, Efrem Violato, Aravind Ganesh

**Affiliations:** 1 Department of Clinical Neurosciences, Cumming School of Medicine, University of Calgary, Calgary, AB T2N 2T9, Canada; kaden.lam@ucalgary.ca (K.L.); cody.doolan@ucalgary.ca (C.D.); aganesh@ucalgary.ca (A.G.); 2 Graduate Science Education, Cumming School of Medicine, University of Calgary, Calgary, AB T2N 4N1, Canada; 3 Centre for Advanced Medical Simulation, Northern Alberta Institute of Technology, Edmonton, AB T5G 2R1, Canada; efremv@nait.ca

**Keywords:** remote ischemic conditioning, stroke, neuroprotection, pre-hospital care, usability testing, ambulance, emergency medical services, medical device, paramedic, simulation

## Abstract

Background/Objectives: In acute stroke, often-prolonged hospital transport times present an opportunity for early interventions to salvage brain tissue. Remote ischemic conditioning (RIC), where brief cycles of ischemia–reperfusion in a limb are induced to protect the brain, is a promising treatment for this setting. We assessed the usability of a novel RIC system in a simulated emergency response scenario. Methods: Paramedics were asked to use the RIC device in an emergency stroke care and ambulance transport simulation, overseen by a confederate. Feedback on device use was collected through questionnaires, including the System Usability Scale (SUS) and the NASA Task Load Index (NASA-TLX), and a semi-structured interview. Questionnaire responses were summarized using descriptive statistics; interview transcripts were analyzed thematically. Results: Nine paramedics (including the confederate) participated, with a mean of 10.0 ± 10.3 years of professional experience. Questionnaire responses indicated high device usability (mean SUS score: 85.3 ± 12.9 out of 100) and low task-related demands, effort, and frustration (mean NASA-TLX domain scores: ≤3.9 out of 20). Seven paramedics stated they would use the device in daily practice. They expressed concerns related to display screen clarity, interference with standard procedures, cable management, device fragility, and patient discomfort. Suggested improvements included adding indicators of device performance and refining the cuff design. Conclusions: While the device was considered easy to use, paramedics also identified important areas of improvement. With a small, localized study sample, our findings are primarily applicable to the refinement of the RICovery system for use in future clinical trials in the same healthcare setting. However, feedback on the importance of mitigating potential interference of newly introduced procedures with those already established, robustness of equipment, and effective paramedic–patient communication may also help inform the design of other pre-hospital interventions.

## 1. Introduction

In acute stroke, “time is brain” [1]. As stroke progresses, neural tissue is rapidly and irreversibly lost, rendering the effectiveness of acute treatments highly dependent on their timely administration. To meet the need for urgent medical care in the interest of improving patient prognosis, pre-hospital “code stroke” protocols have been widely endorsed, comprising the highest priority dispatch of emergency medical services (EMSs), pre-hospital notification, and rapid transfer to the closest stroke-ready center [2,3]. As supported by current evidence, implementation of “code stroke” systems is associated with shorter onset-to-admission times and door-to-needle times, as well as a higher rate of reperfusion treatment administration [3,4,5,6]. However, the beneficial impact of protocol improvements is contingent on the availability of resources and proximity to stroke centers.

Many patients continue to incur pre-hospital delays, particularly in countries like Australia and Canada, where due to geographic realities hospital transport may take an hour or more [7,8,9]. To address this issue, there is currently only one form of pre-hospital treatment available, which is providing thrombolysis—intravenous administration of a clot-dissolving drug—in mobile stroke units (MSUs) [10,11]. However, widespread implementation of MSUs is hindered by costs and local regulatory barriers [12]. Moreover, in the pre-hospital setting, there are no alternative acute treatments available for patients who are ineligible for thrombolysis.

In this context, remote ischemic conditioning (RIC) presents as a promising, inexpensive, non-invasive and viably administered treatment option [13,14,15]. It involves inducing brief cycles of ischemia–reperfusion through inflation and deflation of a cuff around a limb to protect a remote, vital organ, including the brain [14,16]. Although the mechanisms underlying the neuroprotective effect of RIC are not yet fully understood, animal model studies indicate the activation of humoral, neural, and genetic and inflammatory pathways, in turn promoting cell survival and repair, while inhibiting apoptosis and inflammation [16]. Randomized controlled trials (RCTs) in acute stroke have consistently demonstrated the safety of RIC [13,17,18], with some of the available evidence pertaining to hemorrhagic events. These findings support the potential for early RIC treatment initiation, prior to obtaining results of brain imaging to differentiate between stroke types.

To date, however, research has predominantly focused on RIC use in ischemic stroke, providing some encouraging findings regarding its effect on patient outcomes. Among these is the large, multi-center RICAMIS trial, where compared to usual care, RIC repeatedly administered during a hospital stay was reported to significantly increase the likelihood of excellent functional outcomes [19]. While providing a specific example of RIC use in a pre-hospital setting, a Danish proof-of-concept trial found that after adjustment for baseline perfusion and diffusion lesion severity in a post hoc analysis, RIC delivered during ambulance transport lowered the tissue risk of infarction [20]. However, no significant effect was observed for the primary endpoint of penumbral salvage. Building on this study, the RESIST trial involved initiating RIC in the ambulance and repeating treatment sessions over seven days [18,21]. Significant improvement in functional status with RIC use was revealed in a post hoc, sub-group analysis of patients with stroke due to cerebral small vessel disease and good treatment adherence, yet not in the primary analysis involving all participant data. These mixed results of past studies are met with continuing research interest in understanding the true effects of RIC in a pre-hospital setting, as exemplified by the recently conducted REMOTE-CAT trial [22].

Joining the pursuit to advance investigations into RIC application through a clinical trial program [23], our team recognized that efforts to gain high-quality evidence on this treatment may be compromised by important limitations of existing RIC devices. Identified concerns included (i) a lack of technical support; (ii) the inability to remotely track treatment quality or patient compliance during an intervention, and in turn to facilitate it, as devices needed to be shipped to the lead site for manual upload of data; and (iii) requiring separate sham units, which significantly increases the number of devices needed for a controlled trial. The latter also interferes with blinding; for instance, in trials like RESIST that have used sham comparators, the sham device has been visibly different (e.g., different color). With a view to address these limitations, our team collaborated with an industry partner to develop a novel RIC device, named “RICovery” (patent application: US 17/317771), seeking input from practicing stroke neurologists and persons with lived experience.

Looking ahead to the potential future adoption of RIC treatment, it is relevant to point out that there are no published data on formally involving patients and healthcare professionals in the development or refinement of existing devices. One of the primary purposes of medical technologies is to meet user needs, and incorporating user evaluations in the design process is recognized as critical to ensuring good usability of a new product in the intended context [24,25]. This translates to increased productivity, safety, user acceptance and satisfaction with introduced technologies, and a reduction in use-related errors and need for user training and support [25,26,27].

For usability assessments to help achieve such a development outcome, it is necessary to have real users of a device perform real-life tasks, and record what participants do and say, to then address any uncovered issues [28]. In view of this, the present study aimed to gain insights on the usability of our novel, miniaturized RIC system through its use by paramedics in a simulated emergency stroke care and transport scenario. Received feedback was intended to inform the refinement of the RICovery system and relevant operational procedures in preparation for planned feasibility and efficacy trials.

## 2. Materials and Methods

### 2.1. Study Design, Setting, and Participants

We performed a simulated use study at the Center for Advanced Medical Simulation (CAMS) at the Northern Alberta Institute of Technology (NAIT) in Edmonton, Canada. CAMS is an interactive and interdisciplinary clinical simulation facility that replicates real-world scenarios in clinical, community and home care environments. Testing was conducted over one day in October 2023. The study was approved by the NAIT Research Ethics Board (NAIT REB# 2023-12; approval date: 4 October 2023).

Potential participants were identified from a list of healthcare professionals, interested in participating in research, maintained by NAIT. To gather data from representative end-users, participants of varying knowledge and experience levels, including rural and urban experience, were targeted for recruitment, including practicum-level Primary Care Paramedic (PCP) students, practicing PCPs, and practicing Advanced Care Paramedics (ACPs). Within the practicing PCP and ACP groups, a range of years of experience were sought for inclusion. Participants were invited to participate via email. The sample size for this study was informed by usability consulting project insights, published by the Nielsen Norman Group (experts in usability heuristics) [29,30]. These projects have indicated that a sample of five to eight users provides the optimal return on investment in terms of actionable usability findings discovered to expense incurred. Therefore, we aimed to include eight paramedic users in this study.

All participants gave written, informed consent. Prior to the simulation, paramedics were provided with a standardized briefing on operating the RIC device, including cuff placement and activation of the unit. We intentionally provided minimal information regarding the device to allow an understanding of the ease and intuitiveness of its use. We further provided participants with an overview of what the simulation involves and their role (see Appendix A).

### 2.2. The RICovery Device Prototype

**Figure 1:**
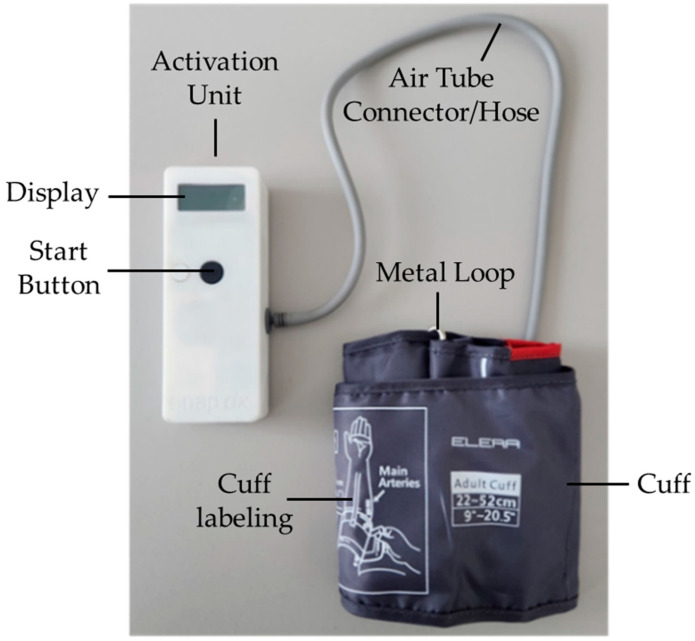
The tested RICovery device prototype.

The RICovery device was developed by SnapDx Inc. and manufactured by FUEL Biomedical, both Calgary-based companies. The tested RICovery prototype, measuring 151 mm × 56 mm × 33 mm, is presented in Figure 1. Its key components include an activation unit with a display screen, a cuff, and a connecting hose. The device allows for delivering standard RIC, consisting of repetitive cycles of cuff inflation to 200 mmHg for 5 min, then deflation for 5 min, for at least four cycles. When in use, the current cycle is indicated on the display screen. As an additional feature, the cuff inflation pressures can be programmed to lower values, so as to suit the tolerance thresholds of individual patients. Treatment records are securely and remotely transmitted through a central hub, allowing quality control and compliance monitoring. Devices are also remotely programmable for “blind” randomization as true or sham RIC. The device is charged through a standard USB cable.

### 2.3. Simulation Scenario

The simulated scenario, detailed in Appendix A, required the transport of a stroke patient by ambulance to a hospital. Participants were randomly assigned to one of two conditions—one with a short (22 min) transport time and one with a long (45 min) transport time. Other than duration, the two scenarios were identical. As paramedics work in teams, a confederate, independent of the research group, accompanied participants during each simulation to maintain fidelity. The confederate did not participate in the use of the RIC device nor did they provide additional instructions regarding device operation, and only acted in response to participant requests. During the simulation, the device was kept in the paramedics’ primary kit. Participants were free to use it in whatever manner they considered appropriate.

A description of the situation was provided to participants at the beginning of each simulation. Briefly, they were dispatched to a stroke call for an elderly patient—an iStan manikin—who was found lying on the floor. With the assistance of the confederate, they were required to transfer the patient to an ambulance simulation box—a reproduction of an actual ambulance, built on a motion platform to replicate the sensation of driving on various road conditions. Participants were alone while providing patient care in the ambulance; however, they could communicate with the confederate over a radio transceiver. Each simulation ended at the allotted time, with a researcher informing the participant they had arrived at the hospital and requesting them to remove the RIC cuff from the patient.

### 2.4. Data Collection and Analysis

All simulations and interviews were audio–video recorded. We used multiple evaluation measures, including observational data, questionnaires, and semi-structured interviews. The confederate contributed to the two latter forms of data collection, separately from other participants, once all simulations had been completed. The confederate data were included as we recognized the unique value of insights gained from involvement in every simulation. For quantitative data collection, we digitally administered the following assessments using the Qualtrics survey software (Qualtrics, Provo UT):

1. The System Usability Scale (SUS)—a standardized 10-item questionnaire that provides a quantitative measure of the usability of a system or technology; each item is rated on a scale from 1 (strongly disagree) to 5 (strongly agree), with responses producing a composite score from 0 to 100 (highest degree of usability);
2. The NASA Task Load Index (NASA-TLX)—a subjective rating scale that measures the perceived demands (mental, physical, and temporal), effort, and frustration experienced during a task, and successfulness in accomplishing it; each item is rated on a scale from 0 (very low) to 20 (very high);
3. A bespoke Device Validation Checklist—a structured instrument assessing seven specific components of the RIC device, rated on a 3-point scale—not acceptable, acceptable but improvements could be made, and acceptable; open text entry boxes were included to allow for further clarification;
4. Additional custom assessment items—five items developed based on the Post-Task Single Ease Questions [31], asking about different aspects of ease of device use on a scale from 1 (very difficult) to 5 (very easy), and two binary (yes/no) questions on liking the device and willingness to use it in daily work.

Observations of participant behavior during simulations and semi-structured interviews were conducted by one researcher, EV. The interview guide included questions regarding general impressions about the RIC device and its look and feel, the experience of using it during the scenario, any encountered challenges or difficulties, anticipated use-related risks, views on potentially using multiple devices, and thoughts about the provided training.

Quantitative data from survey responses were analyzed using descriptive statistics. The scenarios were watched in real time and multiple reviews of the simulation recordings were conducted at a later date. Interview recordings were transcribed verbatim and integrated with notes from observations. The qualitative data were analyzed thematically by BAD, KL and EV. This iterative process involved reading and re-reading transcripts, coding excerpts to capture concepts found in the data, and grouping related codes to develop themes and subthemes.

## 3. Results

Nine paramedics took part in the study, including four providing patient care in the long transport scenario, four in the short scenario, and one consistently serving as the confederate through all scenarios. Paramedic characteristics are summarized in Table 1, including the confederate, who was a 42-year-old male with 12 years of primarily rural professional experience. 

### 3.1. Questionnaire Findings

The results of the SUS and NASA-TLX questionnaires are reported in Table 2. For items included in the Device Validation Checklist, there was no instance of a “not acceptable” response. Of the 63 total responses (7 components × 9 paramedics), “acceptable” was selected 85.7% of the time. The specific components that were indicated as “acceptable but improvements could made” included Attaching the therapy cuff to the limb (N = 4), Cuff inflation cycle (N = 1), Cuff holding at pressure (N = 1), Rest at ambient pressure (N = 1), and Removing the therapy cuff (N = 2). The two main issues mentioned by participants in the open text comments related to the use of the metal loop for placing the cuff and a lack of clarity regarding the current inflation of the cuff, including uncertainty as to whether it was in the inflation or deflation part of the cycle, and no indication of the exact pressure. The confederate, moreover, noted that accidental, premature pressing of the start button resulted in having to detach the cuff from the unit to deflate the cuff, before it could be placed on the patient’s arm.

**Figure 2:**
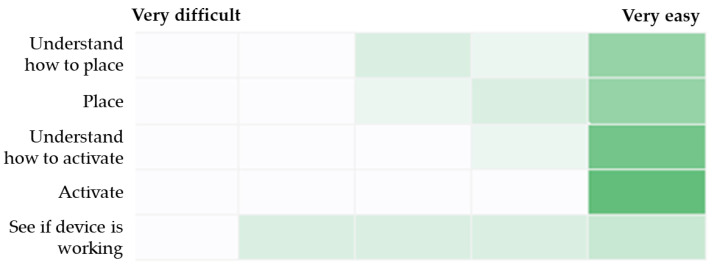
Heat map of responses for ease of understanding and using the RIC device. Note: Darker shade of green indicates a higher frequency of responses for a given scale point.

Responses from the five custom assessment items that addressed ease of understanding and using the RIC device are summarized in Figure 2. All paramedics found that understanding how to activate the device was very easy. The greatest range of responses was recorded in relation to ease of seeing if the device was working, with one participant and the confederate scoring 2/5 (where 5 is “very easy”), two participants scoring 3/5, two participants scoring 4/5, and three participants scoring 5/5. Finally, seven paramedics were “somewhat confident” or “very confident” in the functioning of the RIC device, indicated that they liked using the device, and would use it in their daily practice if it was available to them. Individual participant data for all questionnaires are presented in the Supplementary Materials (Tables S1–S4).

### 3.2. Observation and Interview Findings

Four main themes emerged from the qualitative data analysis. The first theme captured opinions and recommendations regarding the RIC device’s design and way of operating. Here, our findings largely reflected the results of the questionnaires. Paramedics consistently emphasized overall ease of device use. At the same time, however, some participants indicated having difficulties placing the cuff due to the incorporated metal loop, as well as pointing to insufficient clarity of the display screen, causing uncertainty as to whether the device had been working properly. Other concerns related to the device being too fragile to withstand the ‘rough’ handling that is inherent to emergency response situations, and a particularly high risk of the hose becoming detached or damaged. As a solution to a number of identified issues, participants suggested enabling attachment of the activation unit to the cuff. All contributing subthemes are presented in Table 3, alongside a brief description of their key aspects, associated recommendations for improvement, and exemplar quotes.

The second theme, as presented in Table 4, related to views on incorporating use of the RIC device into established workflows, including considerations on device storage, interference with standard procedures, and point of treatment initiation. Participants expressed their desire for specific guidelines on RIC use, with the absence of relevant instructions diminishing their confidence in making decisions. In determining when to begin the intervention, an assumption that RIC treatment is time-sensitive led to placing and activating the device immediately, on scene, as was the case for two paramedics. Most participants, however, initiated RIC in the ambulance—a decision reached either through applying general, established rules of conduct, where other procedures are prioritized over non-life-saving treatment, or due to practical considerations. 

The third theme focused on the anticipated patient experience of the RIC treatment, with implications for the paramedics’ conduct and the patient–paramedic relationship (Table 5). Although, overall, participants did not foresee any life- or health-threatening risks of RIC to patients, many expressed a concern that at least for some the pressure of the cuff would be difficult to tolerate. In such cases, to alleviate patient distress, avoid premature treatment termination, and maintain rapport, participants emphasized the importance of communicating with the patient about the treatment, ‘coaching’ them through it.

The fourth and final theme centered around the paramedics’ need of comprehensive knowledge regarding the RIC treatment, including an understanding of the mechanisms underlying its effect and contraindications. Participants felt that being sufficiently informed was integral to confident device use and RIC treatment decision-making, including how best to embed RIC within established workflows and appropriately respond to any changes in the patient’s status or external circumstances. Moreover, as indicated in Theme 3, the ability to explain the RIC process and its intended benefit was considered important in communicating with patients, and particularly key to promoting treatment adherence and maintaining rapport with those finding the cuff pressure difficult to tolerate. Participants also expressed a concern that without having evidence-based knowledge on the effects of RIC, some paramedics may view it as “just another step in their protocol” rather than an impactful treatment and may not use the device when and as intended.

*I would have liked to know more about why we were using it. Again, that’s […] 50 percent of using anything is the explanation of the patient and being able to answer all those questions. So, in the training, I just would have liked more, more context into why we’re using this. Are there specific cases we’re using this and […] what evidence is there, um, that justifies us using this in that case?*

(P3_930)

*I don’t know exactly how successful these things are at reducing or helping with strokes. Um, if there, if it comes out that they are really successful, maybe that will be a higher priority on my list, but currently it’s kind of an extra thing, as far as I’m concerned*.

(P8_1400)

## 4. Discussion

We conducted a usability study in which paramedics provided feedback on a new RIC device developed for clinical trials in the pre-hospital space (among other settings). Taken together, questionnaire and interview data indicated that the RIC device was easy to use. At the same time, however, participants identified a number of issues that could be improved on to further enhance the RICovery system’s usability. Concerns regarding device design and operation were primarily focused on the clarity of the display screen, risk of damage to the unit and hose, and cuff design incorporating a metal loop. Additional issues were raised around certain aspects of workflow, such as deciding on the point of treatment initiation and interference with standard procedures, as well as the patient experience of RIC, potentially entailing a need to address treatment-related distress.

Our findings provide valuable insights on the usability of the RICovery prototype, which correspond to three of four dimensions of device development distinguished by Tsai and colleagues [24]. With no feedback directly applicable to organizational characteristics, these encompass considerations regarding (i) user action, including user needs, training and personal characteristics (e.g., need to understand mechanisms of RIC to allow confident device use); (ii) technical capability, including user interface design, troubleshooting specifications, and labeling (e.g., clarity of performance indicators on the display screen); and (iii) use environment, including intended location, usage time, and necessity of specific safety and hygiene measures (e.g., stretcher transfer involving high risk of dropping device or detaching hose).

Regarding the latter, challenges inherent to emergency response situations and ambulance settings have also been recognized in previous research on system use. For example, in a study evaluating the usability of a mobile telestroke platform in the United States, it was found that the motion of the ambulance led to instability of system hardware, entailing a need to change and additionally secure its initial position [32], while in a Swedish study, the authors identified that the stress and time pressure associated with responding to a major incident posed a barrier to using a detailed triage tool [33]. The same investigation also revealed a concern that having to record information on triage cards competed with providing medical care for the patient. The issue of interference with other medical procedures was also raised in our study, particularly in relation to the cuff obstructing IV access and hindering blood pressure measurement. 

Unsurprisingly, these between-study comparisons highlight that even in the similar setting of pre-hospital care, each specific system or technology entails a distinctive set of challenges. These, moreover, may need to be addressed differently depending on particular organizational characteristics [34]. As such, for the potential standard application of RIC systems in an ambulatory setting to be successful, it is necessary to expand the currently limited evidence on device use in this context. 

### 4.1. Strengths and Limitations

To our knowledge, this is the first study to formally evaluate the usability of an RIC system in a pre-hospital setting, given that paramedic feedback had not been published from the two relevant trials conducted in Denmark [18,20]. Important strengths of our research included use of a systematic usability testing framework with structured questionnaires (including validated assessments of usability and task load), detailed feedback interviews with experts, and the fact that a party independent of the device inventors conducted the simulations in an environment that sought to closely replicate real-world transport conditions for the paramedics. Therefore, the feedback provided from participants was less likely to be biased or influenced by external stakeholders.

One limitation was that all our usability testers were from the same region and therefore our findings may not generalize to other geographical locations with different organizational characteristics, standard operating procedures, and available resources of emergency medical services. Secondly, it is important to note that we had not involved real patients at this stage of our research. While participating paramedics shared their views on the anticipated patient experience of RIC, and its potential impact on the paramedic–patient relationship and treatment adherence, these issues are yet to be formally investigated. The study limitations emphasize the need for future feasibility trials to comprehensively account for the impact of real-world, patient and environment-specific factors on the application of the RICovery system, with its usability potentially overestimated in a simulated setting.

### 4.2. Practical Implications of Study Findings

**Figure 3:**
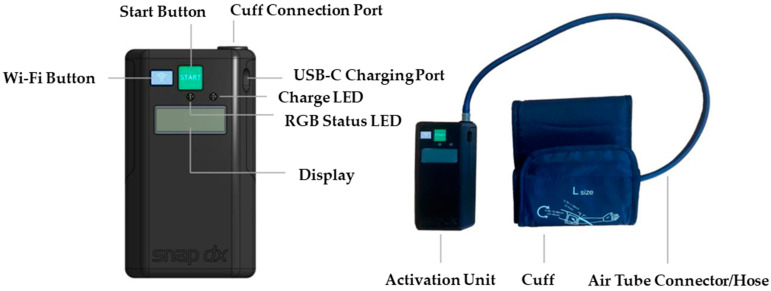
Revised RICovery prototype, guided by the usability testing feedback, that is set for testing in a clinical trial.

While some concerns raised by paramedics will require more discussion, such as addressing interference with other medical procedures, those regarding the device’s design have already been incorporated into the prototype’s revisions. Based on participant feedback, the blood pressure cuff has been replaced with one that does not have a metal loop and only consists of Velcro. Furthermore, the activation unit case has been upgraded to a sturdier and more robust material. Lastly, the information shown on the device screen has been updated to provide more clarity (e.g., device indicates what cycle it is currently on, the percentage of inflation achieved as inflation progresses, and the time left in the therapy cycle). A status LED (light-emitting diode) has also been added that indicates the device state (green for inflating, yellow for deflating). This revised prototype, presented in Figure 3, is now set to be tested in a pilot study with acute stroke patients in a stroke ambulance environment (clinicaltrials.gov registration: NCT05967728).

Assuming that future trials will indicate that this RIC intervention is feasible and effective in improving stroke outcomes, it appears that the use of the refined device could be implemented during patient transport without significantly increasing demands on paramedics. The ease with which the treatment can be initiated (with a single button press) and flexibility regarding where the cuff can be applied (either to the upper or lower limb) seem particularly advantageous in this context. Prior to implementing the intervention in daily practice, paramedics will require brief training on the RICovery system. Our findings suggest that successful RIC treatment delivery does not require a high level of background knowledge or experience of device use. Nonetheless, it will be important to incorporate information on how the intervention is intended to work, elaborating on the underlying mechanisms and contraindications for use, to support paramedics in confident treatment decision-making.

## 5. Conclusions

Through advancing the development of a novel RIC system, this study constitutes a step towards addressing an unmet need for efficacious pre-hospital stroke treatments. While the device was considered easy to use, paramedics also identified important areas for improvement. With a small, localized study sample, our findings are primarily applicable to the refinement of the RICovery system for use in future clinical trials in the same healthcare setting. However, feedback on the importance of mitigating potential interference of newly introduced procedures with those already established, robustness of equipment, and effective paramedic–patient communication may also help inform the design of other pre-hospital interventions.

## Figures and Tables

**Table 1:** Participant characteristics (N = 9).

Age, mean (SD; range)	35.6 (11.3; 21–57)
Sex, N (%)	
Female	5 (55.6)
Male	4 (44.4)
Certification, N (%)	
Primary Care Paramedic	5 (55.6)
Advanced Care Paramedic	4 (44.4)
Years of professional experience, mean (SD; range)	10.0 (10.3; 0–32)
Primary Area of Work, N (%)	
Urban	5 (55.6)
Rural with or without urban	4 (44.4)
Prior usability testing experience, N(%)	
Of any medical device	2 (22.2)
Of an RIC device	2 (22.2)

**Table 2:** Descriptive statistics of responses from the SUS and NASA-TLX questionnaires.

	Mean (SD) (N = 9)	Range (N = 9)
**SUS**	85.3 (12.0)	67.5–97.5
Would Use Frequently	4.0 (1.5)	2–5
Found Device Complex	1.2 (0.4)	1–2
Device Was Easy to Use	4.9 (0.3)	4–5
Would need Support from Technical Person	1.1 (0.3)	1–2
Functions Were Well Integrated	3.8 (1.4)	1–5
Device was Inconsistent	2.2 (0.8)	1–4
Most People would Learn to Use Device Quickly	5.0 (0.0)	5–5
Found Device Cumbersome	1.3 (0.7)	1–3
Felt Confident Using Device	3.9 (1.3)	1–5
Needed to Learn a Lot Before Using Device	1.3 (0.5)	1–2
**NASA-TLX**		
Mental Demand	0.9 (0.8)	0–2
Physical Demand	1.0 (0.9)	0–2
Temporal Demand	3.6 (5.3)	0–15
Effort	0.9 (1.3)	0–4
Frustration	3.9 (6.3)	0–20
Performance	13.4 (8.0)	0–20

System Usability Scale (SUS); NASA Task Load Index (NASA-TLX).

**Table 3:** Detailed overview of Theme 1: RIC device design and operating.

Subtheme	Elucidation/Key Points	Related Recommendations	Exemplar Quotes
Ease of device use	Opinion that the RIC device was easy to operate and/or that providing the treatment was a low-demand task	Not applicable	*[…] in terms of actually ease of use—no issues at all. It’s basically the same as a blood pressure cuff. So, we do that every day. There is literally one button to press, so it’s mental load is very low. Um, I didn’t feel like it interfered with my treatments.* (P1_800) *[…] it’s very simple to use. It’s not something I had to continually pay attention to, which I liked*. (P7_1330)
Device reliability and monitoring	Statements and observations indicating participants’ uncertainty as to whether the device was functioning as intended.	Improving the clarity of the device display, including: a larger screen; use of color indicator lights (e.g., green when working correctly, red if error occurs); providing information on the cuff’s inflation status.	*It was hard to see the screen. It was the only thing that I found difficult and then there wasn’t anything, like, I think throughout it might have worked a couple times, but then it just kind of stopped working, so, and then I wasn’t sure if it was gonna, like, supposed to work or not. I don’t know*. (P2_830)
Cuff placement challenges due to metal loop	Statements and observations indicating issues with placing the cuff due to the incorporated metal loop. The loop reduced users’ freedom of movement and in some situations introduced a challenge of having to unthread and rethread the cuff.	Switching to a single contiguous Velcro cuff, as is currently used by paramedics for blood pressure monitoring.	*I wish the cuff was like the ones we have on blood pressures that […] you don’t have to run the straps through a loop to go back around. Just cause then like, I had already put the IV on, I put the lead on for my ECG and then I had to, I went to just slide the cuff over, I went, ‘oh, I can’t do that’, so I had to open the cuff, put it on, run it through that [loop]*. (P8_1400)
Activation unit placement	Statements and observations indicating variability and uncertainty regarding placement of the activation unit in the ambulance so as to avoid any damage to the device. With limited free surface area, some participants placed the unit on the patient, yet considered this as potentially disrespectful and disagreeable to the patient.	Enabling attachment of the activation unit to the cuff or integrating the two components. Making the device hose longer.	*I wish the cord was a little longer maybe, just cause, I had to set it on the patient or beside. Like, I feel like in the real world I wouldn’t want to set it on the patient, I guess I could, but probably want to set it bedside, so the longer cord might be helpful just so it’s not hanging off of them*. (P8_1400)
Cable management	Identifying the risk of the RIC device hose becoming detached from the activation unit, pinched, or severed. Monitoring for this was considered an additional attentional demand alongside watchfulness of other standard equipment cables.	Enabling attachment of the activation unit to the cuff or integrating the two components. Making the device hose shorter.	*I don’t know if that would be better. It’s a lot of cables running and, um, cable management is important to me, but I know, especially when I was new, My cables were all over the place and then you’re getting them caught in wheels, you’re getting them caught when you’re lifting and lowering, um, they fall, they become, they unhook, those kind of things*. (P3_930)
Device robustness	Concerns regarding the robustness of the device. Given the circumstances in which paramedics work, accidents involving the device were viewed as inevitable.	Constructing the activation unit casing of more durable materials, making it more resistant to damage and preferably waterproof.	*We tend to drop things in puddles, in the snow, or lose them, leave them on scenes. I know, they fall out of a bag or something like that*. (P3_930)
Unintentional device activation	Identifying the risk of unintentionally pressing the activation button.	Turning the device on and off should require a longer press of the button. Adding a protective cover over the activation button.	*[…] that on-off button is quite sensitive, so it’s easy to accidentally bump it. And then it’s on, but then you turn it off by accident, as it’s, you’re trying to move the patient, and it’s kind of just getting in the way.* (Confederate)
Device labeling	Overall opinion that the labeling on the device was simple and easy to understand, although certain issues were identified, including a nonobvious indication of the direction in which the cuff should be applied and potential to confuse the RIC device with a blood pressure monitor.	Adding a “This side to patient” label on the cuff. Adding an apparent label or distinct coloring so the device would not be confused with a blood pressure monitor. Adding step-by-step instructions on how to apply and operate the device on the back of the activation unit.	*And you see that on a blood pressure cuff as well too, it says this side to patient, and it shows like, how to put the cuff on, written on the cuff…* (Confederate)
Device size and weight	Overall opinion is that the device is advantageously small and light, supporting ease of transfer and storage. Some participants suggested a slightly higher weight could be beneficial, assuming an association with improved durability and being treated with more care and respect by users.	Potentially making the device slightly heavier.	*[…] the size is good. It would fit in lots of different kits. I was able to fit in my cargo pocket*. (Confederate) *I wasn’t sure if I was going to break it if it dropped. I’m not sure if it’s fragile or not*. (P5_1200)
Cuff sizes	Recognizing that the one provided cuff size may not be suitable for all patients, particularly those who are obese.	Providing multiple, different sized cuffs with the device.	*[…] I feel like if someone is quite overweight or edematous that’s going to make it harder to put it on the lower extremity because you’ll need like a bigger cuff if that’s possible too to have a variety of cuff sizes*. (P1_800)

**Table 4:** Detailed overview of Theme 2: Implementation of RIC into emergency response conditions and workflow.

Subtheme	Elucidation/Key Points	Related Recommendations	Exemplar Quotes
Optimal storage	Opinions regarding where the device should be stored when not in use, which related to defining the timepoint of treatment initiation. Suggestions included storage in theprimary kit for initiating treatment on scene/as soon as possible or in the monitor case for initiating treatment in the ambulance.	Not applicable	*I’d probably keep it just down, I wouldn’t put it on top of my kit, because I feel like a stroke patient, it’s not something I go to every day, right, so I’d put it probably somewhere where I know where it is, but I’d have to maybe pull, maybe under the IV kit or something, just so it’s, I know where it is quickly to grab it, but it’s not like the first thing I’m going to grab, right*. (P8 _1400)
Decision-making around RIC initiation	Lack of specific guidelines related to uncertainty in participants’ decision-making. Two users applied the device on-scene and six in the ambulance. Factors considered in making this decision included the following: Assumed time-sensitiveness of treatment (only factor favoring on-scene initiation); Prioritizing life-saving procedures, linked to avoiding delay in hospital transport; Following the Vital Signs, Oxygen, Monitor, IV, Treatment (VOMIT) protocol; Practical consideration, including risk of damaging device and/or detaching hose during stretcher transfer.	Providing evidence-based guidance as to when the RIC treatment should ideally be initiated.	*Um, I didn’t, honestly, it didn’t even pop into my mind, like, I know it was there to use the device, but again, my priority is getting the patient to the hospital because that’s, like, the life saving treatment. […] It would have been something that I think would be nice to know, like, do we, is it an on-scene thing, or is it a, you know, in transport? Um, once we got into the back, I think it, because obviously knowing it’s a study, I was like trying to prioritize getting it on sooner rather than later.* (P1_800)
Interference with other procedures	Concerns about RIC interfering with the execution or effects of other procedures, including blood pressure monitoring, establishing bilateral IV access, and delivery of IV drugs.	Providing evidence-based guidelines regarding simultaneous placement of an IV and RIC cuff, and IV drug delivery with RIC. Placing the RIC cuff on lower rather than upper limbs.	*If it needs to be on the unaffected arm, then it would be difficult to do serial blood pressures and run IVs. Because the IV also has to be on the unaffected arm*. (P6_1230)
Use of multiple RIC devices	Most participants found this feasible and were willing to use multiple devices if this translated to increased benefit to the patient. Some, however, expressed concern for excess task demands, having to activate and monitor multiple devices and manage more cables.	Enabling attachment of activations unit to the cuffs or integrating the two components. Having a single activation unit for multiple cuffs.	*I think you would definitely need more hands in the back if you’re going to be applying multiple. But if this can be done en route, very easily. Um, yeah, then I don’t see, I think it would just take time*. (P7_1330)

**Table 5:** Detailed overview of Theme 3: Anticipated patient experience of RIC and implications for the patient–paramedic relationship.

Subtheme	Elucidation/Key Points	Related Recommendations	Exemplar Quotes
Concerns over patients’ tolerance of RIC	Anticipating that for some patients (particularly elderly) the cuff may cause bruising or skin tears and induce discomfort that is difficult to tolerate. This in turn could lead to increased patient fear, confusion, volatility or aggression, refusing the RIC treatment, and jeopardizing rapport between the patient and paramedic.	Placing the RIC cuff on a lower rather than upper limb (assumed to be better tolerated). Having the option to inflate the cuff to a pressure lower than 200 mgHg.	*[…] multiple times I’ve had patients that don’t like the blood pressure cuff and they’re pulling at it and pulling at it and pulling at it. Where they’re like, ah, this hurts! And then they escalate. And then, you know, it might be something like that. That, like, the patient just getting frustrated and exploding because of the pain that it’s causing. And then the medic trying to calm him down, calm him down, explain why they’re doing it, and then it gets in the way of the relationship*. (Confederate)
Strategies for improving patient adherence	Emphasizing the importance of communicating with patients about the treatment and maintaining a positive relationship to help manage discomfort and improve adherence.	Displaying information on the cuff’s inflation status to inform patients about the stage of treatment progress. Providing paramedics with comprehensive information on RIC to facilitate answering patient questions and providing reassurance, and conveying anticipated benefits of treatment adherence.	*So, with a, a stroke it would probably stay away with from like sedative agents and things to calm them down. So it would probably be pain management. I, having experience with, with taking blood pressures with seniors, or patients, especially with high cuff pressures, it is uncomfortable in tourniquets, so usually it’s just some calming measures and pain control. […] Especially when they know the risks and the benefits, so it’s really an understanding, it’s an education.* (P6_1230)

## Data Availability

Quantitative data collected during the study are provided in the Supplementary Materials. Interview transcripts are not publicly available due to the potential risk of identifying participants from their content but are available from the corresponding author on reasonable request.

## Supplementary Materials

The following supporting information can be downloaded at https://www.mdpi.com/article/10.3390/neurolint16060105/s1: Table S1: Individual user responses on the System Usability Scale; Table S2: Individual user responses on the NASA Task Load Index; Table S3: Individual user responses on the Device Validation Checklist; Table S4: Individual user responses on Custom Assessment Items.

## Appendix A

Pre-Simulation Briefing (or “Prebriefing”) read to participants in the ‘briefing’ room:

1. Treat the following simulation as a real 911 ambulance call.
2. You are operating under AHS Medical Control Protocols.
3. You’ll be functioning as one-half of a Basic Life Support ambulance.
4. You have been provided with a RIC device. We ask that you use the device when providing care. Please use the device in any way that you see fit.
5. What you see in the scenario is what you get. If you need more resources or information, ask for it.

The situation: You are dispatched to a “28 Charlie” (Stroke/CVA) for an elderly patient at NAIT. The caller stated that they were talking with the patient and suddenly they started to slur their speech and talk funny. The caller thinks the patient may be having a stroke. The closest hospital with a stroke center is 15 min (45 min) away. There are no stroke ambulances available. You will need to transport the patient.

Scene One: The patient is laying on the floor when EMS arrives.

The onset time was 15 min ago. The patient is a custodian at NAIT and was cleaning up a classroom and conversing with a colleague. The patient’s colleague on scene witnessed the event and stated that while the patient was sweeping, they suddenly dropped their broom. The colleague helped the patient to lay down because they were worried they would fall. The patient now has left sided body weakness and slurred speech. Obvious left facial droop is noted. No trauma seen.

V/S—alert and oriented × 2 (confused to time and place), GCS 14, HR—88 bpm, BP—188/100, Sp02—95% on room air, RR—16, BGL—3.7 mmol/L, Temp—36.2 C, ECG—sinus rhythm, LAMS score—5 (facial droop—1, grip strength—2, arm strength—2).

Scene Two: The paramedics and patient are in the ambulance.

The participants should transport the patient to the ambulance. The stroke symptoms remain (LAMS 5) regardless of treatment. The patient remains confused, with slurred speech etc. The transport will last 15 or 45 min, depending on the condition. Participants will provide care as they see appropriate.

Repeat V/S—LOC—GCS 15 (a + o × 4 if D50W administered), HR—70 bpm, BP—210/100, Sp02—96% on room air, RR—16, BGL—8.2 mmol/L (if 12.5 g of D50W administered doubled if more is given), Temp—36.2 C, ECG—sinus rhythm, LAMS score—5 (facial droop—1, grip strength—2, arm strength—2).
