# Supplementary material for: Evaluating the Usability of a Remote Ischemic Conditioning Device for Pre-Hospital Stroke Management: Insights from Paramedic Simulations
Source: Neurol Int. 2024 Nov 9;16(6):1405–20. doi: 10.3390/neurolint16060105 (PMC11587117; doi:10.3390/neurolint16060105)
Supplement: Supplementary file 1 [file neurolint-16-00105-s001.zip › neurolint-3225985-supplementary.pdf]

SUPPLEMENTARY MATERIALS: Usability testing of a novel remote ischemic conditioning system for pre-hospital stroke care.

Table S1. Individual user responses on the System Usability Scale.

	P1	P2	P3	P4	P5	P6	P7	P8	Confederate
Total	92.5	92.5	95.0	97.5	67.5	97.5	75.0	72.5	77.5
Would use frequently	5	5	5	5	2	5	5	2	2
Found device complex	1	1	1	1	2	1	2	1	1
Device was easy to use	5	5	5	5	5	5	4	5	5
Would need support from technical person	1	1	1	1	1	1	2	1	1
Functions were well integrated	3	5	5	5	1	5	3	3	4
Device was inconsistent	1	2	2	2	2	2	3	2	4
Most people would learn to use device quickly	5	5	5	5	5	5	5	5	5
Found device cumbersome	1	1	1	1	1	1	2	3	1
Felt confident using device	4	5	4	5	1	5	4	3	4
Needed to learn a lot before using device	1	1	1	1	1	1	2	2	2

Note: Items rated on a scale from 1 (strongly disagree) to 5 (strongly agree).

Table S2. Individual user responses on the NASA Task Load Index.

	P1	P2	P3	P4	P5	P6	P7	P8	Confederate
Mental demand	1	0	2	0	1	0	1	1	2
Physical demand	1	0	2	0	1	0	2	1	2
Temporal demand	1	0	2	0	10	1	15	1	2
Effort	1	0	1	0	1	0	4	1	0
Frustration	1	3	1	0	20	0	5	4	1
Performance	15	17	20	20	1	20	10	18	0

Note: Items rated on a scale from 1 (very low) to 20 (very high).

Table S3. Individual user responses on the Device Validation Checklist.

	P1	P2	P3	P4	P5	P6	P7	P8	Confederate
Attaching therapy cuff to limb	3	3	2	3	2	3	2	3	2
Pressing gray start button	3	3	3	3	3	3	3	3	3
Cuff inflation cycle	3	3	3	3	3	3	3	3	2
Hold at therapy pressure	3	3	2	3	3	3	3	3	3
Cuff deflation cycle	3	3	3	3	3	3	3	3	3
Rest at ambient pressure	3	3	2	3	3	3	3	3	3
Removing therapy cuff	3	3	2	3	2	3	3	3	3

Note: Items rated on a scale where 1 indicates not acceptable, 2 – acceptable but improvements could be made, and 3 – acceptable.

Table S4. Individual user responses on Custom Assessment Items.

	P1	P2	P3	P4	P5	P6	P7	P8	Confederate
How easy was it to understand how to place the device on the patient?	4	5	5	5	5	3	5	3	5
How easy was it to place the device on the patient?	5	5	4	5	3	5	5	5	4
How easy was it to understand how to activate the device?	5	5	5	5	5	5	5	5	4
How easy was it to activate the device?	5	5	5	5	5	5	5	5	5
How easy was it to see the device was not working?	4	3	2	5	5	5	4	3	2
Liked using the device	Yes	Yes	Yes	Yes	No	Yes	Yes	Yes	Yes
Would use the device in daily work	Yes	Yes	Yes	Yes	No	Yes	Yes	Yes	No

Note: First 5 items rated on a scale from 1 (very difficult) to 5 (very easy).
